# Supplementary material for: Liquid-Phase Dehydration of Glycerol to Acrolein with ZSM-5-Based Catalysts in the Presence of a Dispersing Agent
Source: Molecules. 2023 Apr 8;28(8):3316. doi: 10.3390/molecules28083316 (PMC10143559; doi:10.3390/molecules28083316)
Supplement: Supplementary file 1 [file molecules-28-03316-s001.zip › molecules-2266524-supplementary.pdf]

Supplementary Materials for

Liquid-Phase Dehydration of Glycerol to Acrolein with ZSM-5-Based Catalysts in the Presence of a Dispersing Agent

Lin Huang, Bo Wang,* Licheng Liu and Armando Borgna
Institute of Sustainability for Chemicals, Energy and Environment, Agency for Science, Technology and Research, 1 Pesek Road, Jurong Island, Singapore 627833, Singapore

1. Preparation of P-ZSM-5

P-ZSM-5 (H_3PO_4-modified H-ZSM-5) included 2 %P-ZSM-5_280 , 10 %P-ZSM-5_280, 15 %P-ZSM-5_280 and 20 %P-ZSM-5_280. They were prepared by impregnation. In a preparative experiment, a total of 2 g of H-ZSM-5 was impregnated with a 15-85 % H_3PO_4 solution at 22 °C. After 3 h of standing, the impregnated sample was dried under vacuum at 22 °C for 2 h followed by drying at 120 ◦C for 12 h. Finally, the resulting sample was calcined at 500 ◦C in N_2 for 5 h to give P-ZSM-5.

2. Deconvolution of NH_3-TPD Curves of Solid Acid Catalysts

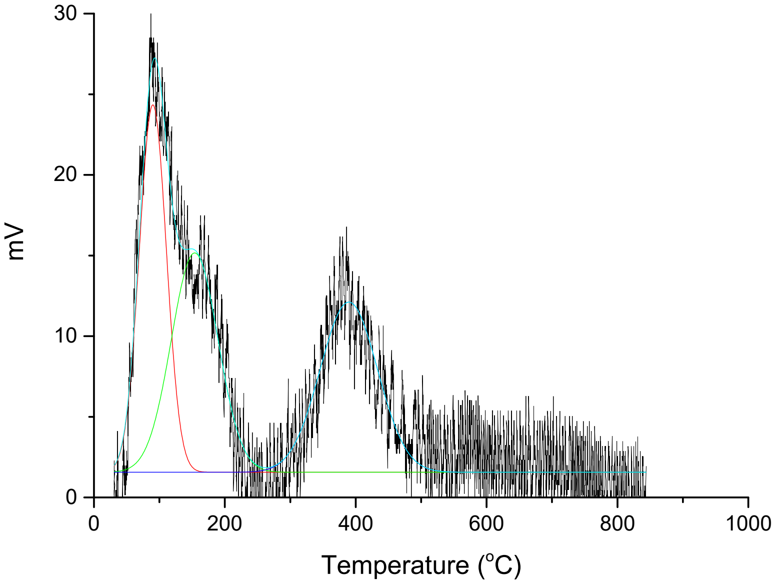

Figure S1. NH_3-TPD of ZSM-5_280.

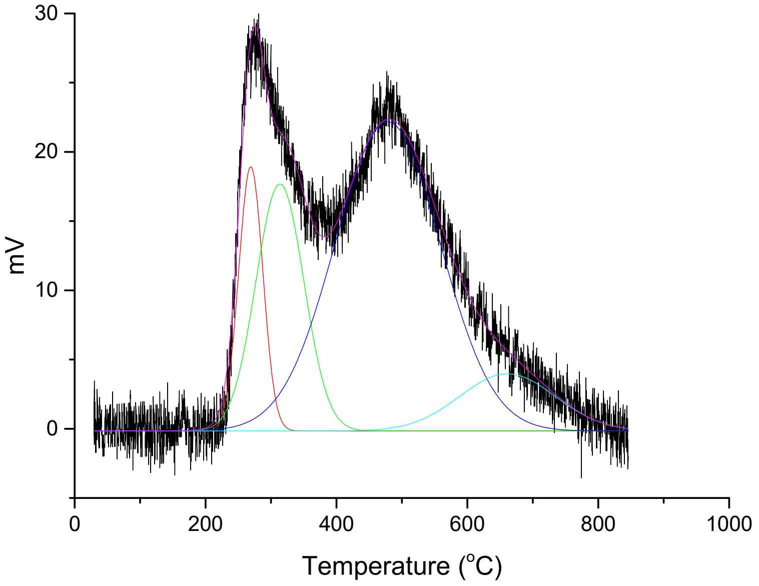

Figure S2. NH_3-TPD of ZSM-5_80.

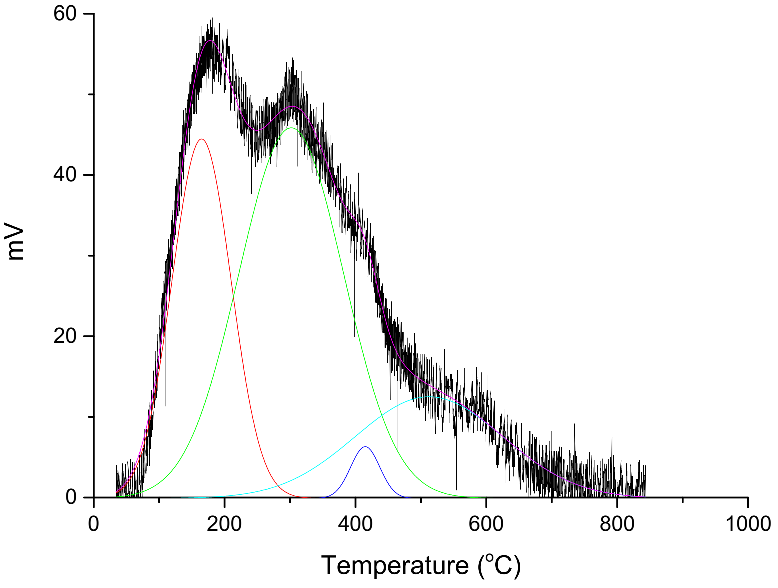

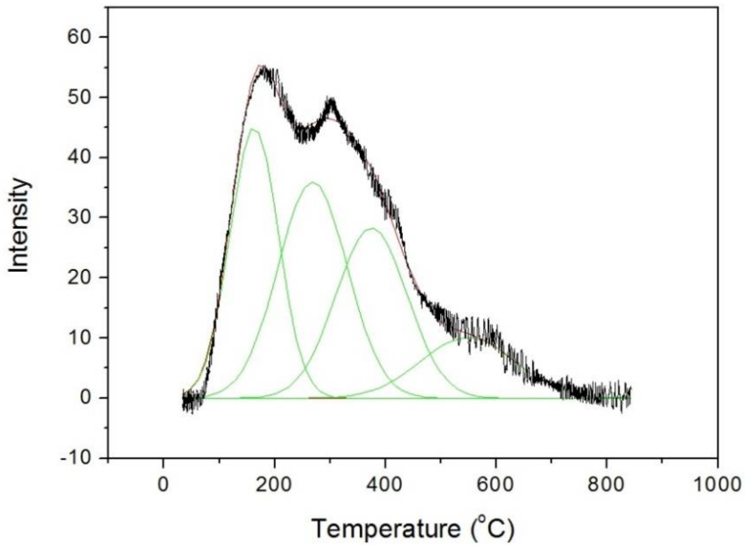

Figure S3. NH_3-TPD of 2 %P-ZSM-5_280.

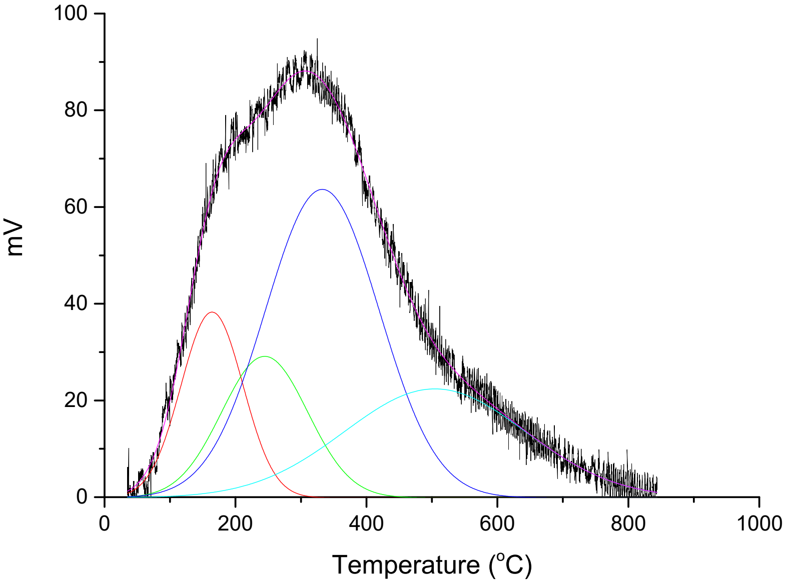

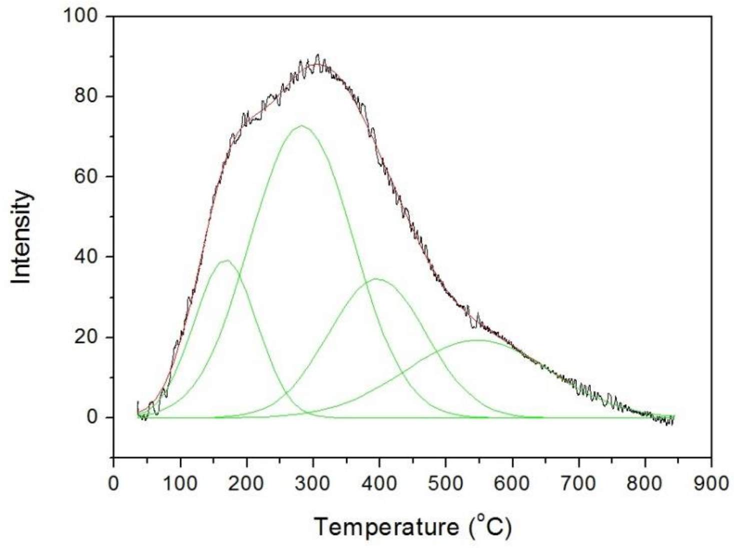

Figure S4. NH_3-TPD of 10 %P-ZSM-5_280.

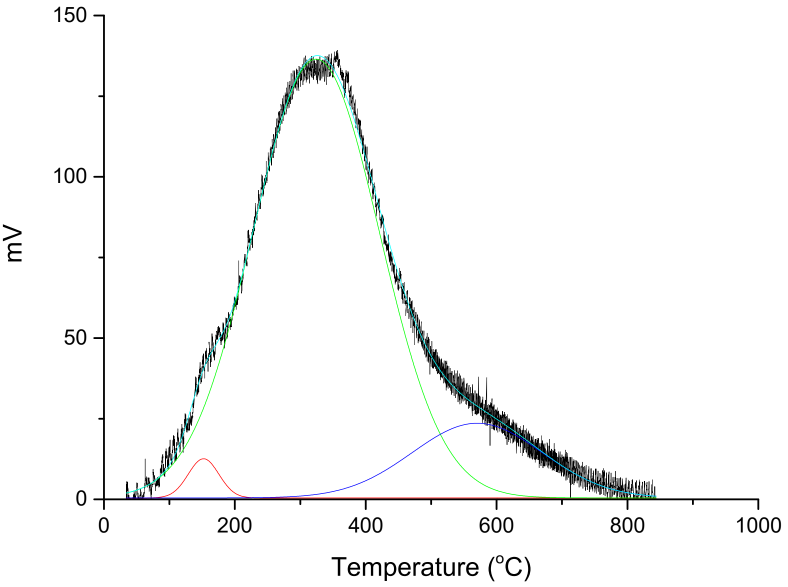

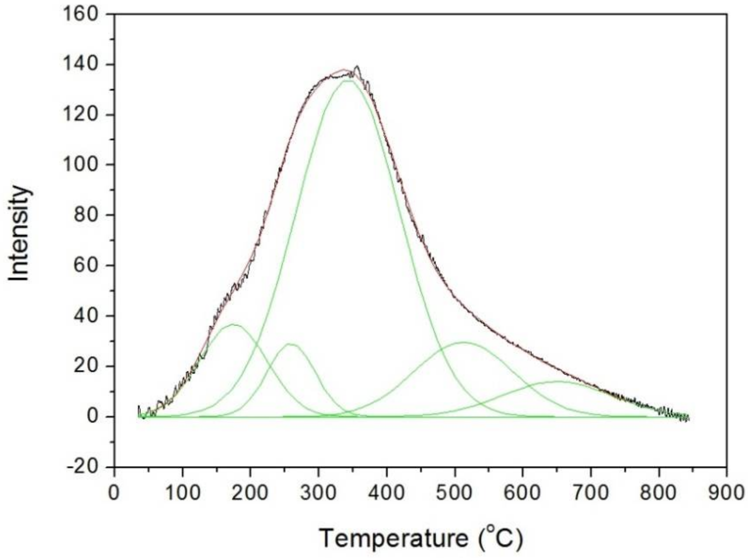

Figure S5. NH_3-TPD of 20 %P-ZSM-5_280.

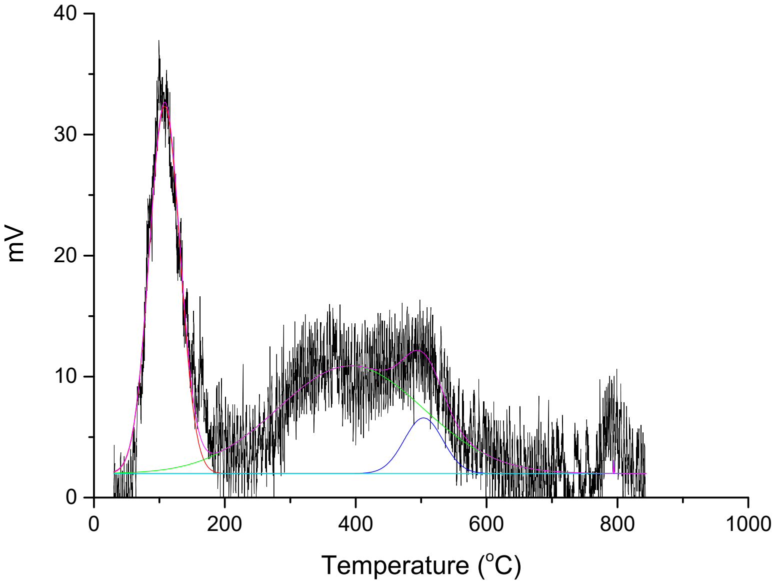

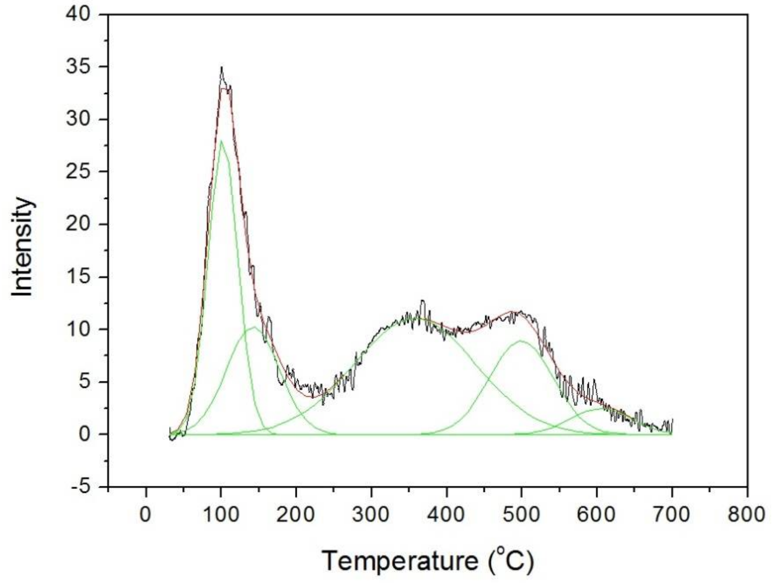

Figure S6. NH_3-TPD of CsPW.

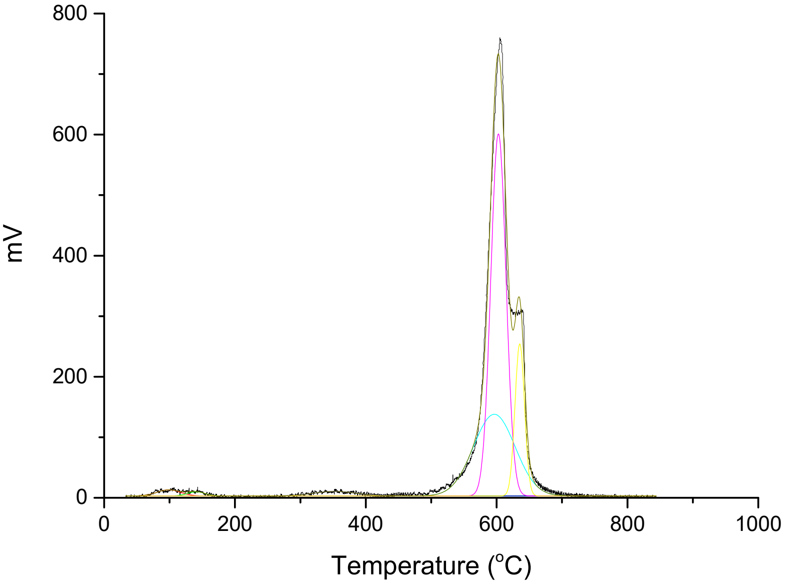

.

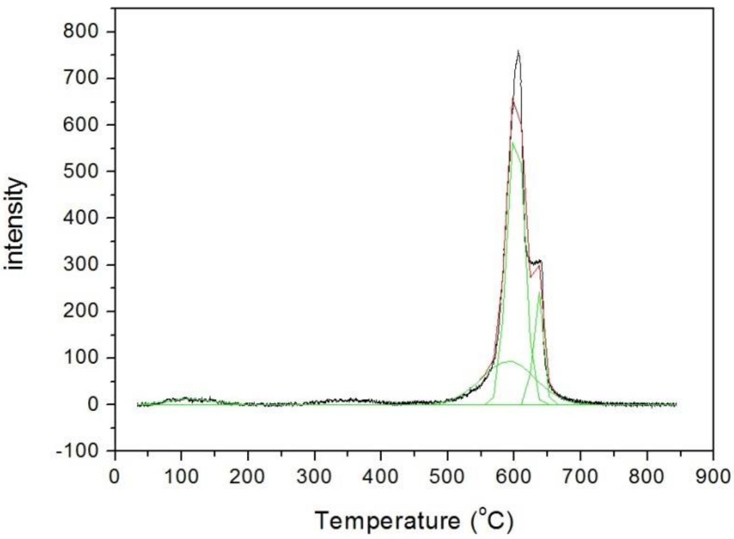

Figure S7. NH_3-TPD of HPW.
